# Through their eyes: A retrospective mixed-methods study on the experiences and support needs of children growing up with a parent with Huntington’s disease

**DOI:** 10.1177/18796397241304333

**Published:** 2024-12-19

**Authors:** Maud MJ Daemen, Annelien A Duits, Lucienne B van der Meer, Ruben L Andriessen, Ruth B Veenhuizen, Renske Wassenberg, Tanja Peeters, Lia de Jager, Mayke Oosterloo

**Affiliations:** 1 Department of Psychiatry and Neuropsychology, Mental Health and Neuroscience Research Institute, Maastricht University, Maastricht, The Netherlands; 2 Department of Medical Psychology, Radboud University Medical Center, Nijmegen, The Netherlands; 3 Department of Medical Psychology, Maastricht University Medical Center, Maastricht, The Netherlands; 4 Department of Clinical Genetics, Leiden University Medical Center, Leiden, The Netherlands; 5 Department of Neurology, Mental Health and Neuroscience Research Institute, Maastricht University Medical Center, Maastricht, The Netherlands; 6 Huntington Expert Center Atlant, Apeldoorn, The Netherlands; 7 Huntington Expert Center Land van Horne, Weert, The Netherlands; 8 Huntington Expert Center Topaz Overduin, Katwijk, The Netherlands

**Keywords:** Huntington's disease, children, needs, mixed-methods, support, family system

## Abstract

**Background:**

Growing up with a parent with Huntington's disease (HD) profoundly impacts children. However, this impact and children's needs are often misunderstood, even by professional services. Even when resources are available, children often feel that their needs are unmet, raising concerns about the adequacy of available guidance and support.

**Objective:**

This study aims to offer an in-depth understanding of the multifaceted impact of growing up with a parent with HD, examining the needs for professional guidance on emotional and social aspects, and identifying specific areas where support can be improved to better aid them.

**Methods:**

This retrospective study utilized an exploratory sequential mixed methods design, combining qualitative focus groups (*n *= 13) and a quantitative survey (*n *= 23). Qualitative data were analyzed using an inductive thematic analysis with a descriptive phenomenological approach. Quantitative data were analyzed using descriptive statistics.

**Results:**

The impact of HD on children extends across various domains, affecting self-development, social interactions, and family dynamics. Support received at home varied, with limited access to professional help. Support needs primarily revolved around emotional support and access to comprehensive information. Key support providers, such as parents, peers, mentors, healthcare providers and coaches with expertise in HD, play crucial roles in addressing these needs.

**Conclusions:**

The study underscores challenges faced by children in HD families. By centering our efforts on the emotional well-being of these children, offering tailored information, involving their social network, providing community-based support, and strengthening parental support systems, we can improve the support required by children in these families.

## Introduction

Huntington's disease (HD) is an incurable neurodegenerative disease that carries a 50% risk of being inherited genetically. It typically manifests between 30 and 50 years of age,^1^ a time in life where persons often raise children. Children raised by a parent with HD can be confronted with the progressive deterioration of their parent's physical, psychiatric, and cognitive functioning. For example, they may perceive changes in personality, mood, initiative, and a reduced empathic understanding.^2,3^ Such changes, along with the relatively high prevalence of adverse experiences such as parental dysfunction, psychiatric problems or domestic violence, may have a profound impact on a child's development.^4^ Children raised by a parent with HD are more prone to developing a poor attachment style in adulthood, and they show an increased risk of psychological and relational problems later in life.^5^ Furthermore, children can feel uncertain whether they have inherited the disease. Balancing hope and fear may be a continuous psychological burden to cope with.^6^

As the progression of HD unfolds, the dynamics between the affected parent and their children undergo significant changes. Children may take over tasks and responsibilities from their parents due to the limitations of their parent with HD and due to the reduced availability and capacity of the other parent.^7–11^ Due to the progressing disease of the parent, family conflicts, disrupted family dynamics and social isolation may occur.^8,12^ Experiences with both parents and other affected family members may shape teenagers’ current decisions and future plans.^13^ Amidst these dynamics, open communication about the disease and social support becomes crucial. It has been found to help children and parents better cope with and admit the implications of the disease.^7,10,14^ Particularly if children learn about HD from an early age, they seem to cope better.^10^ Nevertheless, children often hardly talk about their situation with others, and they frequently experience a lack of support from their social circles.^8,9^

Prior research indicates that the impact of HD on children is often underestimated, even by professional services.^15,16^ Also, children express difficulties in finding or accessing the guidance they need.^17^ Even when resources are available, children often feel that their needs for understanding, care, and support are unmet, which raises concerns about the adequacy of available guidance and support.^8^ Moreover, parents often serve as gatekeepers to such support. Their denial or lack of awareness about HD not only obstructs access to necessary support, but also limits others’ comprehension of the home situation, making it difficult for others to help children involved.^15^

To address the challenges of children raised by a parent with HD, and to prevent future problems, it is imperative that we investigate how to provide adequate care for these children. Notably, there is a lack of research focusing on the experiences of children in the Netherlands within this context. Therefore, the current study explores the multifaceted impact of growing up with a parent with HD, examining the needs for professional guidance on emotional and social aspects, and identifying specific areas where support can be improved to match their needs.

## Methods

### Study design

This study employs a retrospective design because people can reflect on experiences that occurred before they turned 18 and identify what might have been helpful to them. They may find it challenging to recognize these aspects while they are still in the midst of the experience, particularly if they are very young. Additionally, a retrospective design allows for a better understanding of the entire period up to their 18th year, covering a longer timeframe. This retrospective study utilized an exploratory sequential mixed methods design to gain an in-depth understanding of the impact and support needs of children who grew up with a parent with HD in the Netherlands. A mixed methods design was chosen because it yields complete results and integrates qualitative and quantitative data, to gain a comprehensive understanding of issues.^18^ Qualitative data was gathered through focus group discussions. Focus groups were chosen to discuss and explore various experiences. Participants can respond to one another, which potentially uncovers deeper insights or new topics that individuals might not consider on their own. Additionally, participants may feel more comfortable sharing their experiences with peers who have had similar experiences, as focus groups can be less intimidating than individual interviews.^19^ Focus groups prove to be particularly valuable in sensitive research as they encourage more sensitive and personal disclosures and reveal themes that might not emerge in individual interviews.^20^ Subsequently, a survey was created based on the qualitative findings to collect quantitative data. An integration of quantitative results was used to follow up on and test the qualitative findings. An ethical non-waiver was obtained from the Medical Ethics Committee of Maastricht University Medical Center, the Netherlands (#2022-3308).

### Participants

Participants were recruited through the Dutch HD Association, specialized HD centers in the Netherlands, and the healthcare professionals involved. An information leaflet was distributed, and recruitment messages were posted on the social media of the Dutch HD Association. Inclusion criteria for participants were: being 18–35 years of age; having a parent with HD; having experienced HD symptoms of their parent before 18 years of age; and speaking Dutch as their primary language. Four people were excluded from the focus groups due to being over 35 years of age. For the survey, all who applied to participate met the inclusion criteria.

### Data collection and procedure

All eligible participants were included in the study after informed consent. A total of three online focus groups were conducted. All focus group were audio recorded and guided by an experienced moderator (MO or AAD) and an assistant (MMJD). None of the moderators had a clinical relationship with the participants or their parents. Topics were selected from a collaborative brainstorming session with all researchers, considering prior literature and the potential for deeper exploration. Questions were carefully designed to encourage equal participation and thoughtful discussion. The semi-structured topic list (Supplemental Table 1) comprised questions regarding participants’ (retrospective) experiences while growing up with a parent with HD, the (subjective) impact of these experiences on their personal and family life, experiences with social and professional support and participants’ current reflections thereof, and participants thoughts on what they believe would have been beneficial or desirable when growing up with a parent with HD. The focus groups lasted between 90 and 120 min. Audio recordings were transcribed verbatim.

A survey was designed to test or confirm the qualitative findings gained from the focus groups. This survey was sent to several participants in the focus groups to verify whether the topics aligned with the content of the focus groups or whether topics were missing. Multiple categories of questions were formulated that primarily focused on the impact of growing up with a parent with HD, experiences with help and support, and advice and support needs. Questions aimed at frequency or intensity were measured on a 5-point Likert scale ranging from 0 “Never” to 4 “Always”. Statements were assessed on a 5-point Likert Scale ranging from 1 “Strongly disagree” to 5 “Strongly agree”. The other questions were asked using yes/maybe/no answer options or a point-scale ranging from 1 to 10. Completing the survey took approximately 20 min.

### Data analysis

All transcripts of the focus groups were uploaded into Atlas.ti Version 23.2.0 for data analysis. Two authors (MMJD and RLA) independently conducted an inductive thematic analysis, using a descriptive phenomenological approach to prioritize the lived experiences and to acquire a profound comprehension of their realities.^21,22^ The authors began with multiple readings of the transcripts to become familiar with the data. Subsequently, they openly coded the data and identified higher-order themes. These themes and categories were further deliberated upon with the research team, to eventually refine and finalize the themes. In case of any discrepancies, they were addressed through discussion to ensure trustworthiness of the study findings. Finally, the authors who conducted the analyses verified that all data had been coded, no codes were assigned without corresponding meaningful data, and data saturation had been reached with no new information emerging from the analysis.^23^ The results are reported in adherence to the Consolidated Criteria for Reporting Qualitative Research (COREQ),^24^ and served as input for the quantitative survey. Four focus group participants registered for the survey, but since the survey data is anonymous, it cannot be traced back to any individual or considered in the interpretation of the survey results.

Descriptive statistics (frequencies, mean and standard deviation) were calculated using SPSS Statistics version 27.

## Results

A total of 16 persons participated in three focus groups and 23 persons participated in the survey (response rate 85%). Three participants from the focus groups were excluded from the analysis, because their parents’ symptoms started after they turned 18. Table 1 shows an overview of participants’ characteristics. Data analysis revealed three main themes and corresponding subthemes reflecting the experiences and support needs of children growing up with a parent with HD (Table 2).

**Table 1.** Participant characteristics.

Variable		Focus groups (*n *= 13)	Survey (*n *= 23)
Gender	Male	1	8
	Female	12	15
Mean age (SD)		26.5 (7.0)	27.1 (6.1)
Parent with HD	Father	6	11
	Mother	7	12
Age of the participant when HD symptoms started in their parent (SD)		10.5 (5.3)	10.5 (4.1)
Age when participant was informed about their parent's HD (or risk of getting HD)		Not asked	11.6 (5.4)
Family composition	Only child	1	4
	One sibling	7	10
	Two siblings	3	6
	Three siblings	2	3
Living situation when growing up (period up to the age of 18)	With both parents living together in one house	11	19
	With both parents until a certain age, then with one parent	2	4

**Table 2.** Main themes and corresponding subthemes.

Main theme	Subtheme
Experiences and impact of growing up with a parent with HD	Impact on the individual and self-development Personal risk for HD Family dynamics in the shadow of HD Interactions in social environments Long-term reflections on impact of HD
Experiences gained with help and support	Support received at home Support in social environments Interactions with professionals
Advice and support needs	Personal needs throughout the experience with HD Key support providers Pillars of support

### Experiences and impact of growing up with a parent with HD

#### Impact on the individual and self-development

Qualitative findings showed that growing up with a parent with HD poses challenging circumstances for children, affecting their individual growth and development. Participants indicated they often grappled with low self-esteem, lack of self-confidence, and feelings of loneliness throughout their childhood. The focus on the parent with HD at home led them to suppress their own emotions. Many found themselves in a constant survival mode, resulting in stress, anxiety, and uncertainty. Quantitative findings showed that 50% of participants (*n *= 10) (strongly) agreed they had been unable to develop well due to growing up with a parent with HD.

‘*He [parent with HD] was mainly focused on himself and had no attention for me, which made me feel like I didn't matter.’* – Female, father with HD, participant 5 in focus group 2

The circumstances at home frequently hindered their school performance, as a result of being distracted and having trouble concentrating. Quantitative data validated these challenges, with 55% (*n *= 11) (strongly) agreeing that the home situation negatively impacted their schooling, and 65% (*n *= 13) struggled with making decisions regarding their education. Reflecting on their past, participants expressed they could have made more of their school years.

#### Personal risk for HD

Participants mentioned they experienced concerns regarding their own risk of developing HD. Although genetic testing is often only possible after the age of 18, many were already thinking about the decision to get tested before reaching this age. This is also evident from the quantitative data, which demonstrated that 80% (*n *= 16) of participants (strongly) agreed they were concerned about whether they would develop HD themselves.

Additionally, qualitative findings revealed that concerns about topics such as genetic testing in the future, anticipating test outcomes, and dealing with the test results of siblings were already relevant before age 18. Reasons mentioned for testing included a ‘need to know’, or future family planning. Reasons mentioned for choosing not to undergo testing included a lack of readiness to know, a perceived lack of utility in knowing, or a clear focus on the present moment.

#### Family dynamics in the shadow of HD

Participants mentioned they often took on a caregiving role, and experienced an atypical childhood with increased responsibilities, such as doing household chores, and caring for siblings and their parent with HD.

*‘When something happened, my mother discussed things with me because she couldn't do that with my father anymore. I just had to be a teenager, but I tried to contribute a bit, like taking over my father's role and sorting things out for my brother and sister.’* – Male, father with HD, participant 3 in focus group 1

**Figure 1.**
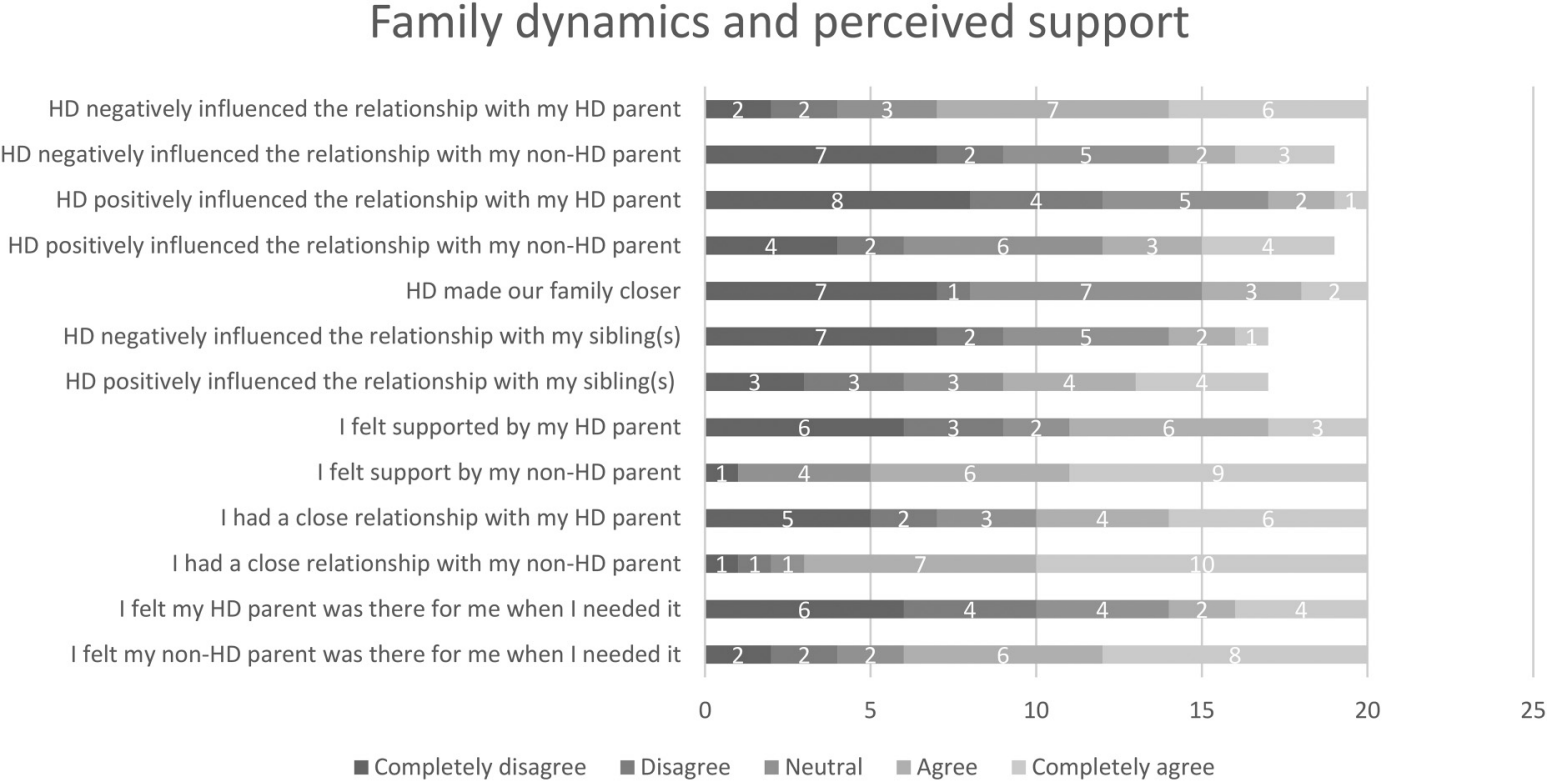
Variations in dynamics and perceived support in HD families.

Quantitative data showed that a total of 53% (*n *= 11) (strongly) agreed that they felt responsible for the health of their parent with HD, while this percentage increased to 76% (*n *= 16) for the non-HD parent's health. Their relationship with the parent with HD generally deteriorated; communication became more challenging, attachment weakened, and distance grew. The impact on the relationship with the non-HD parent varied, with some feeling closer and others sensing more distance due to perceived unavailability. Figure 1 shows the quantitative data on variations in family dynamics.

The numbers represent the frequency of the answer option being filled out in the survey.

Coping with the increasing severity of symptoms became progressively harder, as well as distinguishing the parent from the disease (HD). The symptoms they most noticed in their parent with HD were chorea, problems with planning daily life, perseverative thinking or behavior, delayed information processing, and irritability (Supplemental Figure 1). The symptoms that participants found most challenging to cope with were chorea, anger and aggressive behavior (verbal), irritability, difficulty walking and frequent falling, and lack of initiative (apathy) (Supplemental Figure 2).

#### Interactions in social environments

Qualitative data showed that participants approached interactions with people in their environment in different ways. Some shared details with others to foster understanding and involved their parent with HD in activities. Others kept their situation private due to shame or fear of misunderstanding. A common challenge participants faced was the difficulty in explaining the impact of their family situation and the limited knowledge about HD in their surroundings. Quantitative data showed that 65% (*n *= 13) (strongly) agreed they felt a lack of understanding from those around them, and 57% (*n *= 12) expressed feelings of shame.

Furthermore, when asked if participants avoided sharing details about their home situation due to fear of negative reactions, 43% (*n *= 9) (strongly) agreed, and 33% (*n *= 7) (strongly) disagreed. Regarding the fear of losing friends by sharing such details 29% (*n *= 6) (strongly) agreed, and 53% (*n *= 11) (strongly) disagreed. Of the participants, 29% (*n *= 6) (strongly) agreed they could entrust someone in their surroundings with their thoughts, concerns, or emotions, while 48% (*n *= 10) (strongly) disagreed.

#### Long-term reflections on impact of HD

Participants in the focus groups mentioned they now realize the lasting impact of growing up with a parent with HD. Their perception of ‘normal’ shifted early on, and their boundaries of what they accepted and did for their parents were consistently pushed.

‘*It might sound weird, but I got used to everything. What seemed normal kept changing for me.* – Female, father with HD, participant 4 in focus group 2

They said their sense of self was impacted by the experience of feeling different from peers and being an exception. This was underscored by quantitative data showing that the majority (75%) (*n *= 15) (strongly) agreed they felt different from their peers. According to the participants in the focus groups, the impact remained and had implications for them later in life, influencing how they form relationships, how they raise children themselves, and how they make choices for their future. They all agreed that discussing these matters and receiving support at a young age would promote emotional processing of experiences and would facilitate open communication about HD in later years.

### Experiences gained with help and support

*Support received at home.* While some participants could openly talk about HD, others perceived it as a secret or a taboo subject. Quantitative data showed that 38% (*n *= 8) (strongly) agreed there could be open communication about HD at home, 33% (*n *= 7) were neutral, and 29% (*n *= 6) (strongly) disagreed. Almost half (*n *= 9) (45%) indicated that it was difficult to talk about HD at home. However, 70% (*n *= 14) (strongly) disagreed that discussing HD at home was not allowed and 65% (*n *= 13) (strongly) disagreed that HD was seen as a family secret.

According to qualitative data, most participants faced limited support and a deteriorated bond with their parent with HD, due to emotional absence and a lack of affection. Discussing issues became difficult, especially when the parent was in denial of having HD. Support from the non-HD parent was sometimes perceived as insufficient due to their preoccupation with caring for their partner and working full-time. Others, however, found support and sought advice from their parent on coping with the changing circumstances. Figure 1 shows the quantitative data on variations in perceived support.

#### Support in social environments

Some participants experienced their social environment as a supportive safety net during challenging times, including other family members and friends. Additionally, school was mentioned as an important setting where understanding was experienced, especially from teachers or mentors who were aware of their situation. Other participants felt less understood, leading them to share less with their social circles. This reinforced a negative cycle of limited openness and limited support from others.

‘*Nobody understood our situation. There was a lot of misunderstanding from others. It felt like nobody could help which made me feel sad.’* – Female, mother with HD, participant 3 in focus group 3

Quantitative data emphasized that the perceived support has been insufficient, as 55% (*n *= 11) of the participants (strongly) disagreed that they felt supported in dealing with the situation and changes, 25% (n = 5) were neutral and 20% (*n *= 4) (strongly) agreed. Of the participants, 32% (*n *= 6) (strongly) agreed they received sufficient emotional support and 37% (*n *= 7) (strongly) agreed they received sufficient social support.

#### Interactions with professionals

According to qualitative data, participants mentioned that their unfamiliarity with healthcare services led to difficulties in accessing specialized guidance. As a consequence, they spent a long time searching for appropriate support. Their feeling of not being seen or heard has led to a resistance towards seeking professional help.

*‘I didn’t get help from someone specialized in HD. I experienced a lot of resistance towards professional help, because it felt that they didn’t understand me. After that, I avoided help for a long time.’* – Female, mother with HD, participant 2 in focus group 3

They also indicated that healthcare services were geographically distant, and the hurdle of traveling long distances for specialized help was challenging.

Quantitative findings validated the challenges highlighted in the qualitative data, confirming that 21% (*n *= 4) (strongly) agreed they received sufficient professional support, while 57% (*n *= 13) missed and would have needed a healthcare professional. Those who accessed professional help had sporadic contact with psychologists or case managers. However, the latter mostly focused on partner support. Participants also had contact with the general practitioner, but their impact in facilitating referrals or providing support was perceived as limited. A total of 40% (n = 8) had contact with the general practitioner (rating 5.8 on a scale from 1 to 10), 40% (*n *= 8) with a psychologist (rating 5.7), 50% (*n *= 10) received guidance from school (rating 7.0), 20% (*n *= 4) from a case manager or social worker (rating 7.5), and 5% (*n *= 1) participated in a youth peer support group (rating 8.0). In all these forms of support, the majority stated that contact occurred ‘rarely’ to ‘sometimes’. Regarding sources of information, participants predominantly used informational websites and rarely used videos, books, or magazines.

### Advice and support needs

**Figure 2.**
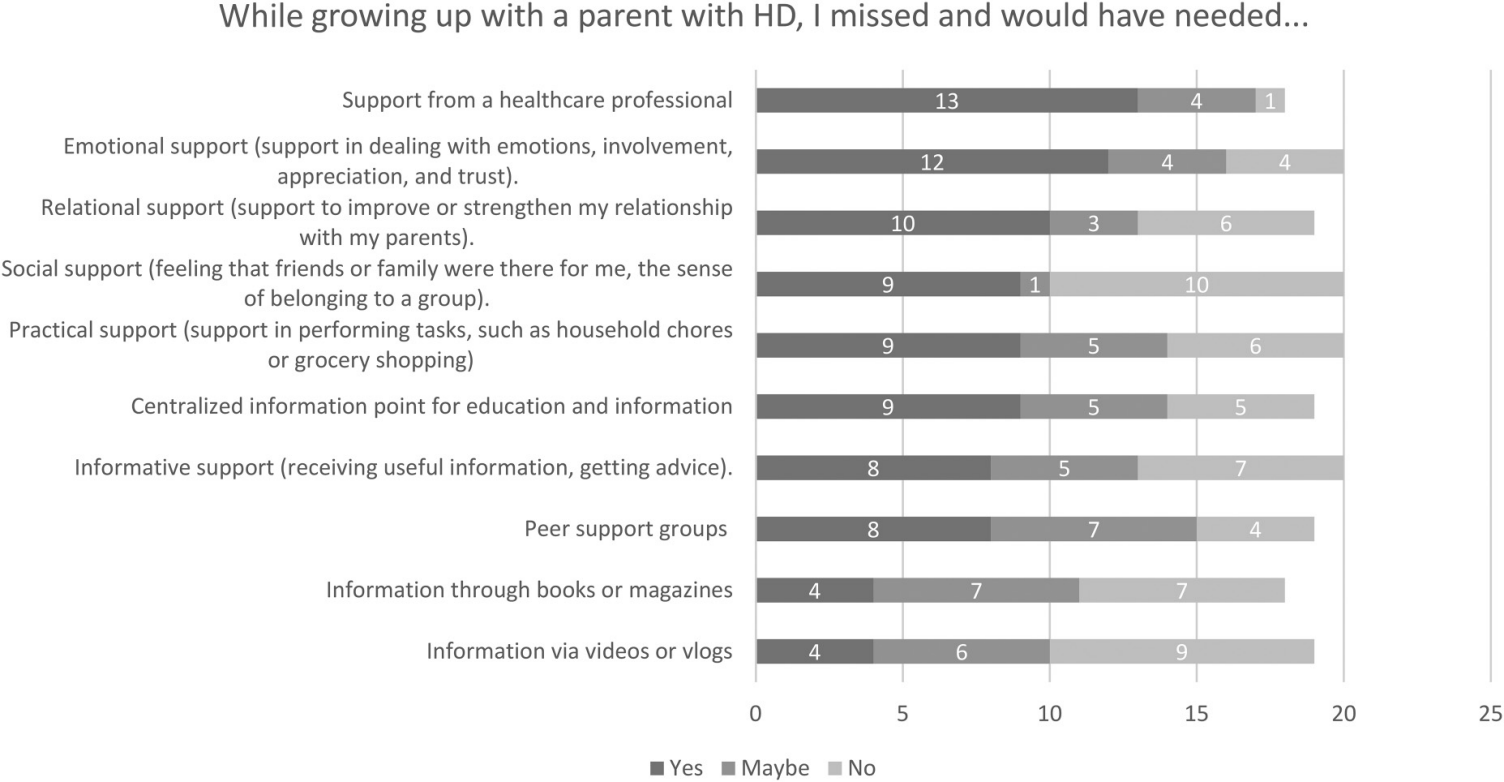
Support needs.

*Personal needs throughout the experience with HD.* Qualitative data revealed that emotional support is one of the most crucial needs; to feel seen, heard, and loved. According to the participants, there should be an emphasis on addressing and discussing matters, especially for children who may not have this opportunity at home. This can be with a healthcare professional, but also in school settings, for example a mentor. Quantitative data supported that participants primarily missed emotional and relational support, while they did have a need for it at that time (Figure 2).

‘*Feeling heard is important. For children who can’t put things into perspective or cannot tell someone at home, that seems very important to me if something can be done about that.’ –* Female, mother with HD, participant 1 in focus group 1

Additionally, there is a need for information, for instance, about what HD entails. This is valuable for both the individuals themselves and their surroundings, enabling them to better explain the impact of their parent's illness to others and fostering greater understanding. Quantitative data revealed variations in the extent to which participants missed or needed informational support. When information is provided, there is a preference for a centralized information point for education and information (Figure 2).

The numbers represent the frequency of the answer option being filled out in the survey.

Guidance on specific matters is also valued. For example, the subject of undergoing testing, as they already grapple with questions on this subject before turning 18. Quantitative data showed that 72% (*n *= 13) (strongly) agreed they would have appreciated support in dealing with concerns about their own risk of ever getting HD. Furthermore, over half of the participants (65%) (*n *= 11) (strongly) agreed that they would have valued support in the period before the onset of HD in their parent.

Additionally, 76% (*n *= 13) of participants (strongly) agreed they would have appreciated greater involvement from their social environment, such as friends being more aware of the situation, providing practical support, or being more supportive in other ways.

#### Key support providers

The parent without HD is primarily in the position to provide emotional support, which, according to the participants in the focus groups, they sometimes lacked. The feeling of always having someone to turn to is highly valued, because they felt they had to constantly adapt to the parent with HD, leading them to neglect their own needs. Contact with peers can be beneficial for sharing experiences, although some found it confronting or uncomfortable if HD was perceived as a secret at home. When asked in the survey if participants would have desired peer support, 42% (*n *= 8) responded with ‘yes’, 37% (*n *= 7) with ‘maybe’, and 21% (*n *= 4) with ‘no’ (Figure 2).

In terms of professional support, qualitative findings showed that preference is mainly given to someone with expertise in HD, which was not always the case in the contacts they had in their childhood. The preference leans towards a confidant, supportive figure, or coach, because seeing a psychologist at a young age was felt as intimidating and might evoke feelings of shame in front of peers.

‘*It's a difficult age. If you say you have to go to a psychologist, classmates say you’re crazy, well, that's at that age. You’ve never really heard of a psychologist at that age, it sounds heavy right away.’* – Female, mother with HD, participant 5 in focus group 3

In the context of support at school, a personal mentor is recognized as an important person to be there for them.

#### Pillars of support

According to the qualitative results, early and continuous monitoring is crucial in organizing support for children in HD families. Professionals need to be attentive, especially when young children are involved. Participants stress the importance of an approach that focuses on both the family and the individual. Support should be available for the entire family, not just the patient and the partner. The participants believe that the extent to which children are involved in caregiving for their parent with HD should be assessed on an individual basis and should carefully be discussed.

They also stressed the importance of care services ensuring that if the parent with HD withholds care, it doesn’t result in a lack of support for the child. Children do not always recognize the need for support, but in retrospect, participants emphasize the importance of support and having the feeling that someone is there for you, sees you, hears you, and talks with you. While recommending active involvement of the social network, they acknowledge difficulties in talking about HD and its impact, especially at a young age.

Additionally, participants suggest providing information at a centralized point for accessing information, and they propose that support and the provision of information should have a positive orientation. Information should be framed in a positive manner and should be focused on exploring possibilities, with the aim of making the time spent with their parent with HD as enjoyable as possible.

## Discussion

The current study delved into the multifaceted impact of growing up with a parent with HD, examining the needs for professional guidance on emotional and social aspects. In this way the study responds to the need to better understand the complex impact of HD on children, to know what they might need and how they might be supported.

Most of the findings about the impact of growing up with a parent with HD correspond with results of previous literature on this topic. Participants experienced low self-esteem, feelings of loneliness, and the focus on the parent with HD at home led them to suppress their own emotions.^2,7,8^ Furthermore, the feeling of obligation to take on parental responsibilities is similar to other publications.^11,13^ As in previous research, participants rarely shared their experiences at school or within their peer group.^2^ They tried to conceal their situation, while at the same time they felt a lack of support from those around them.^9^ Although participants in our study felt reluctant to entrust friends with their thoughts or emotions, the majority was not afraid of losing friends by sharing such details. However, the majority was afraid for negative reactions of misunderstanding. Interestingly, on the one hand 45% of the participants stated that discussing HD openly at home was challenging. On the other hand, 70% disagreed that discussing HD at home was not allowed and 65% disagreed that HD was seen as a family secret. This indicates a clear distinction between feeling that it is difficult to discuss HD openly and feeling that discussing HD is not allowed. Previous research has shown that while families face difficulties in open communication due to stigma, emotional strain, or limitations imposed by the disease itself, they often do not perceive the discussion as forbidden.^11,25^ Open communication is important as it helps children make sense of their experience, feel more in control of their circumstances, and discuss support options.^26^ However, the hereditary nature of HD can lead to family breakdown and create a source of secrets, which hinders communication and limits the sharing of feelings among family members.^27^ Building trust with parents is crucial but can be very challenging.

This study contributes to understanding the needs for professional support for children growing up in an HD family. Most of the participants indicate that professional help is wanted especially regarding emotional and relational support, but also on genetic testing possibilities. Professional help should be provided in a way that is accessible and sensitive, ensuring that children do not feel as though there is something wrong with them. Ideally, this support should be provided by people who have expertise in HD and its impact on family dynamics. This would help ensure that children feel understood and supported. It is also essential to acknowledge that children in HD families may be at an increased risk for a range of psychological issues, such as adverse childhood experiences, anxiety, and depression.^4,10,17^ As such, access to psychological support remains highly important and beneficial for these children. The participants in this study also underscore the significance of school as an important setting for support and understanding, both from peers and mentors. Here we can conclude that also non-healthcare professionals and peers may play an important role in the support of these children. A comprehensive approach that includes both professional and community-based support is valuable for children, as it creates a network of care, offering diverse sources of strength and understanding.

Moreover, our findings underscore the pivotal role of the non-HD parent in providing emotional support. However, HD in families can compromise their availability as a source of support.^11^ This study reveals mixed perceptions among children regarding the support they receive from the non-HD parent. Previous research also confirms that the level of support varies.^2,7,10^ Additionally, the type of support differs, with some parents offering emotional support, while others focus more on instrumental support.^7^ Adopting a family systems approach offers the opportunity to provide comprehensive support aimed at every family member, with early and continuous monitoring as an essential pillar of support. Strengthening the parental role can foster healthy development of the child. Providing self-management support aimed at empowering partners of people with HD in their role, can help enhance their ability to support their children.^28^ Also, increasing the engagement of professionals in encouraging parents to talk openly with their children about the disease holds potential.^29^ In this way, children not only receive the necessary information but also feel seen and understood. Additionally, strengthening parental bonds can potentially enhance the parent-child relationship and foster a supportive family environment.^3,30^

It is essential to acknowledge that the level of external support greatly depends on the family environment. If one of the parents receives support from HD specialized professionals, it is important to explore if professional guidance for the child is needed. Professional support may not be necessary when the parent is open about the disease, able to provide emotional and relational support to the child, and maintains good psychological well-being. In some cases, support from family members, friends, teachers at school, or others from the social environment may be sufficient. When necessary, specialized healthcare organizations can provide information and guidance to informal support providers. Organizations such as the Huntington Disease Youth Organization (HDYO) provide information on how to support children and how to talk to them about the disease. Targeted informational materials are available to educate and empower support providers. In case direct professional care is needed, children strongly prefer healthcare providers with expertise in HD. However, it is important that this support is provided in a setting and role that does not make them feel like they need a healthcare provider because something is wrong with them. The Scottish Huntington's Association (SHA) Youth Service, exemplifies this approach by offering support and age-appropriate information through youth advisors in a non-‘psychology’ setting, including collaboration with schools, for example.

Regarding offered professional care, participants cited unfamiliarity with healthcare services and geographic barriers as obstacles to accessing specialized support. These challenges led to prolonged searches and increased resistance to seek professional help. This reinforces previous findings highlighting children's struggles in accessing adequate support, with many feeling their needs for understanding and care remain unmet.^8,17^ Here we can conclude, that in addition to offering specialized support for children, it is important that this support is easily accessible. Given the complexity of issues such as not being heard and accessing specialized support, comparing the barriers and facilitators in different countries is valuable for future research. This comparison can help provide tailored recommendations suited to diverse contexts. Investing in effective support strategies for children in HD families holds broader societal implications, promoting greater awareness and understanding of genetic conditions.

### Strengths and limitations

The sequential mixed methods design allowed for a comprehensive understanding of children's experiences and support needs. Additionally, the use of a survey enabled individuals who may feel uncomfortable sharing their experiences in an online focus group to still contribute their insights. The retrospective study design allowed for the collection of experiences throughout the entire period of youth and adolescence up to the age of 18. However, there is a risk of recall bias, as the way adults reflect on their childhood needs may be less reliable, for example, because their increased life experience might influence their perspective. To mitigate this, an age limit for inclusion was imposed up to 35 years. It would be interesting for future research to examine what children and young people today report and whether these findings align with the current results. Furthermore, the number of participants who completed the survey is relatively small. Although our results reflect the conclusions in previous research on this topic, we may have missed certain items regarding support methods and needs. For example, no individuals with a migration background participated in this study, so cultural differences and influences were not accounted for. It's worth noting that we made intensive efforts to recruit participants, suggesting a potential barrier to participation and sharing experiences.

## Conclusions

The findings emphasize the significant impact and challenges faced by children in HD families. Despite these challenges, access to specialized support remains limited, with many children feeling unheard or unseen. The study highlights the pivotal role of parental support, particularly from the non-HD parent, in fostering healthy development and mitigating the long-term impact of HD on children. To enhance support, it is important to involve the social network, including peers, teachers, or personal mentors at school, to create a supportive and understanding environment. Additionally, support providers, such as mentors, supportive figures, healthcare providers, and coaches who have expertise in HD are needed to offer tailored emotional support and comprehensive information. Adopting a family systems approach with early and continuous monitoring, combined with both professional and community-based support, can form a network of care that provides diverse sources of strength and understanding for children. These insights provide guidance for innovative support efforts aimed at improving the well-being of children within HD families.

## Supplemental Material

sj-docx-1-hun-10.1177_18796397241304333 - Supplemental material for Through their eyes: A retrospective mixed-methods study on the experiences and support needs of children growing up with a parent with Huntington’s disease Supplemental material, sj-docx-1-hun-10.1177_18796397241304333 for Through their eyes: A retrospective mixed-methods study on the experiences and support needs of children growing up with a parent with Huntington’s disease by Maud MJ Daemen, Annelien A Duits, Lucienne B van der Meer, Ruben L Andriessen, Ruth B Veenhuizen, Renske Wassenberg, Tanja Peeters, Lia de Jager and Mayke Oosterloo in Journal of Huntington's Disease
