# Supplementary material for: Through their eyes: A retrospective mixed-methods study on the experiences and support needs of children growing up with a parent with Huntington’s disease
Source: J Huntingtons Dis. 2024 Dec 19;14(1):93–102. doi: 10.1177/18796397241304333 (PMC12231935; doi:10.1177/18796397241304333)
Supplement: sj-docx-1-hun-10.1177_18796397241304333 - Supplemental material for Through their eyes: A retrospective mixed-methods study on the experiences and support needs of children growing up with a parent with Huntington’s disease [file sj-docx-1-hun-10.1177_18796397241304333.docx]

**Supplemental Material**

**Through their eyes: A retrospective mixed-methods study on the experiences and support needs of children growing up with a parent with Huntington’s disease**

**Supplementary Table 1.** Semi-structured topic list.

| Main topic | Subtopics |
| --- | --- |
| Growing up with a parent with HD | - What are your experiences with growing up with a parent who has HD?   - What emotions or feelings did you experience? - How did your parent's HD affect your life? - How did your parent's HD impact family life? |
| Support and needs | - How did you cope with the situation and your parent's HD?   - What challenges did you face? - Did you receive any help or support?   - If yes, what kind of help or support was it?   - Social support (family members, relatives, friends, neighbors, etc.)   - Professional support - Did this help or support contribute positively to your well-being?   - Why or why not? - Did you feel any help or support was lacking?   - What do you think you needed or would have found helpful? |

**Supplemental Figure 1**. Symptoms children notice in their parent with Huntington’s disease.

**Supplemental Figure 2**. Symptoms children found most difficult to cope with in their parent with Huntington’s disease.

The number on the x-axis shows how frequently the symptom was indicated by the participants of the survey (n=23).
